# Enhanced photoelectrochemical water splitting performance using morphology-controlled BiVO_4_ with W doping

**DOI:** 10.3762/bjnano.8.264

**Published:** 2017-12-07

**Authors:** Xin Zhao, Zhong Chen

**Affiliations:** 1 School of Materials Science and Engineering, Nanyang Technological University, 50 Nanyang Avenue, 639798, Singapore, Singapore

**Keywords:** bismuth vanadate, charge separation, nanostructure, photoelectrochemical water splitting

## Abstract

Nanostructures exhibit numerous merits to improve the efficiency in solar-to-energy conversion. These include shortened carrier collection pathways, an increased volume ratio between depletion layer and bulk, enhanced light capture due to multiple light scattering in nanostructures, and a high surface area for photochemical conversion reactions. In this study, we describe the synthesis of morphology-controlled W-doped BiVO_4_ by simply tuning the solvent ratio in precursor solutions. Planar and porous W-doped BiVO_4_ thin films were prepared and compared. The porous film, which exhibits increased surface area and enhanced light absorption, has displayed enhanced charge separation and interfacial charge injection. Our quantitative analysis showed an enhancement of about 50% of the photoelectrochemical performance for the porous structure compared to the planar structure. This enhancement is attributed to improved light absorption (13% increase), charge separation (14% increase), and interfacial charge injection (20% increase).

## Introduction

Solar hydrogen generation is one of the most promising approaches to create clean energy and to overcome the environmental problems associated with use of conventional fossil fuels. Photoelectrochemical (PEC) water splitting generates hydrogen through chemical reactions assisted by photo-generated electrons and holes in semiconductor materials [1–3]. An ideal semiconductor for PEC water splitting requires a small bandgap to capture enough solar light, a high conversion efficiency, a good durability in aqueous environments, as well as low production cost [4]. Compared with the hydrogen evolution at the semiconductor photocathode, the low efficiency of oxygen evolution at the photoanode poses a great challenge to the water splitting process [5]. As a result, great efforts have been made in developing photoanode materials and optimizing their performance. Monoclinic BiVO_4_ is one of the most promising photoanode materials for PEC water splitting, as it meets most of the requirements. It has a theoretical conversion efficiency of 9.1% with a bandgap of 2.4 eV. Moreover, it also possesses a favorable conduction band potential that is very close to the reduction potential of water, and a proper valance band that is more positive than the water oxidation potential [6–10].

One of the problems associated with BiVO_4_ is its relatively short minority carrier diffusion length, which ultimately affects the solar-to-hydrogen efficiency. Nanostructured materials have been often employed to overcome this limitation [11], as they can shorten the carrier collection distance and increase the volume ratio between depletion layer and bulk. In addition, they also offer a high surface area for chemical reactions and enhance light capture due to multiple light scattering within the nanostructures [11–13]. Many research works have been reported for enhanced PEC water splitting performances using nanostructured BiVO_4_ [5–6,12,14–19]. However, most of them require complex processes. Moreover, there has been no report about a facile process capable of continually adjusting the coating morphology from planar to porous structures for doped BiVO_4_.

In this study, we report the synthesis of a morphologically controlled W-doped BiVO_4_ by simply tuning the composition of the precursor solution. Considering the poor electron conductivity of BiVO_4_, which leads to a poor photoelectrochemical performance (see Figure S1, Supporting Information File 1), we employed tungsten as a doping element because it has a higher valence than vanadium and an ionic radius close to that of vanadium. By changing the solvent ratio, planar and porous nanostructured W-doped BiVO_4_ thin films were prepared. The photocurrent of the porous W-doped BiVO_4_ is ca. 50% higher than that of planar W-doped BiVO_4_. We have analyzed this improvement quantitatively with regard to contributions from light absorption, charge separation and interfacial charge injection. The quantitative analysis provides a powerful insight into the materials potentials and limitations, and is useful for the development of other PEC systems in the future.

## Results and Discussion

**Figure 1:**
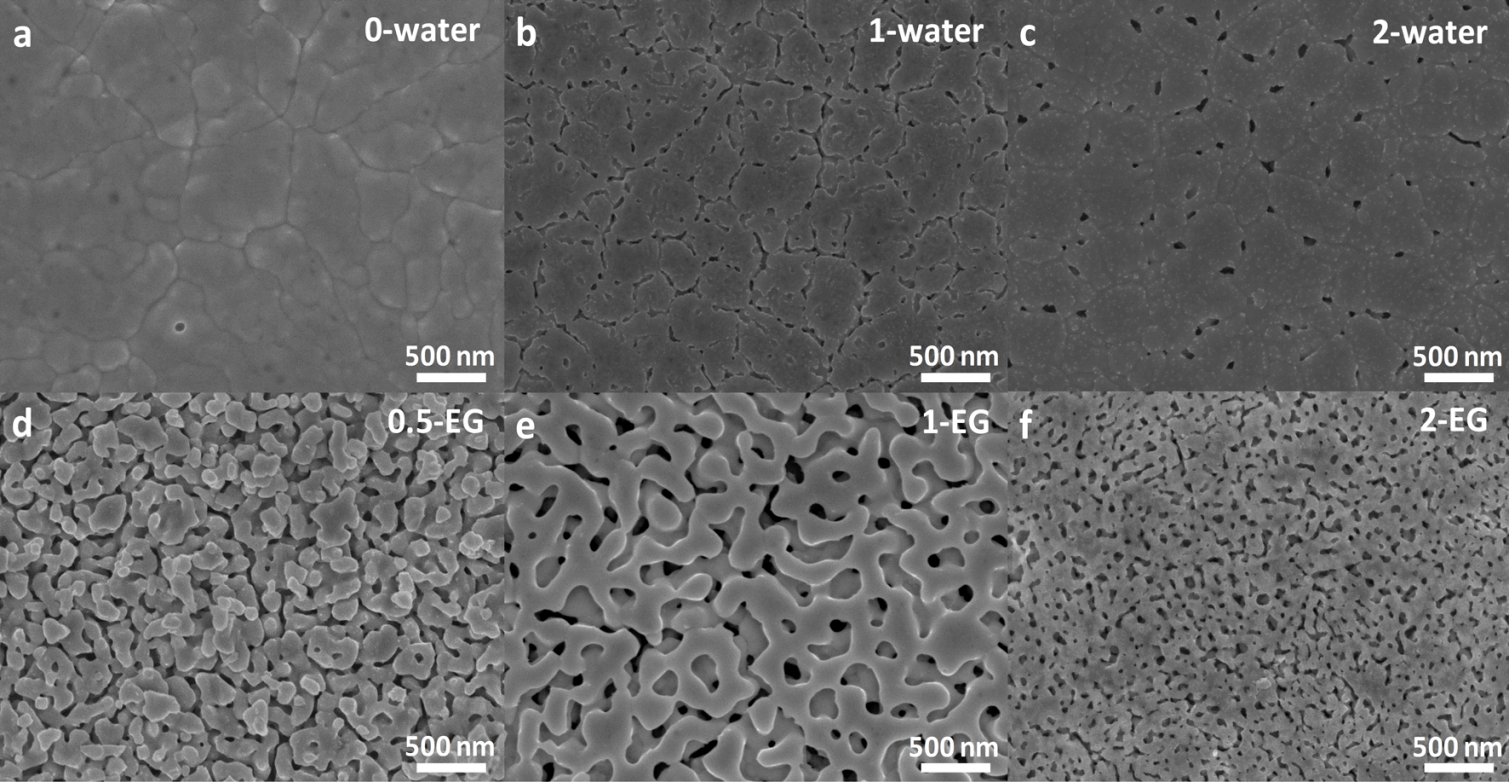
SEM images of W-doped BiVO_4_ thin films with different ratios of water to EG.

The synthesis of W-doped BiVO_4_ thin films was carried out by drop-casting of metal organic precursors with different volume ratios of water to ethylene glycol (EG). The morphologies of the obtained films are shown in Figure 1. An overview of the synthesis conditions and the corresponding sample labels can be found in Table 1. A detailed description of the syntheses can be found in the Experimental section. The films in Figure 1a–c were prepared using EG precursor solutions containing 0.5 mL EG solution of V, 0.5 mL EG solution of Bi, 0.015 mL EG solution of W, and 0.15 mL EG solution of citric acid (CA) to which 2 mL of a mixture of water and EG with different volume ratios was added.

**Table 1:** Elaboration of all samples with different precursors and water-to-EG ratio.

sample	0.1 M Bi EG solution (µL)	0.1 M V EG solution (mL)	0.1 M W EG solution (mL)	1 M CA EG solution (µL)	water (mL)	EG (mL)

0-water	0.500	0.485	0.015	150	0	2.0
1-water	0.500	0.485	0.015	150	1.0	1.0
2-water	0.500	0.485	0.015	150	2.0	0

	0.1 M Bi water solution (mL)	0.1 M V water solution (mL)	0.1 M W EG solution (mL)	1 M CA water solution (µL)	water (mL)	EG (mL)

0.5-EG	0.500	0.485	0.015	150	1.5	0.5
1-EG	0.500	0.485	0.015	150	1.0	1.0
2-EG	0.500	0.485	0.015	150	0	2.0

As shown in Figure 1a–c, the films prepared have a largely planar structure. With increasing water content, small pores emerge with a diameter of ca. 50 nm. Films prepared using water precursor solutions (Figure 1d–f) show nanoporous structures. With increasing EG content, the pore size decreases. The film prepared from the water-based solution with the smallest amount of EG (0.5-EG) exhibits cracks in the film (Figure S2, Supporting Information File 1) and has poor adhesion to the substrate.

The sol–gel method allows reagents to be mixed at the atomic/molecular level, which can increase the reaction rate and, thus, is very suitable for homogenous doping. In this study, it was successfully employed in preparing W-doped BiVO_4_ films with the following sequences. First, a mixture of cations is formed with the aid of an organic complexing agent, citric acid and EG solution. Second, the cations are chelated and form a polymeric resin when dried. Finally, this resin decomposes at high temperatures and forms the targeted W-doped BiVO_4_ films [20]. Jaramillo et al. reported that the ambient humidity affects the rate of both hydrolysis and polycondensation reactions and solvent evaporation, which causes a morphology change of the BiVO_4_ films [18]. We assume that, in our study, the mixture of precursors from different (water or EG) solutions affects the chelate formation and polycondensation, which has great influence on the morphology of the obtained films.

**Figure 2:**
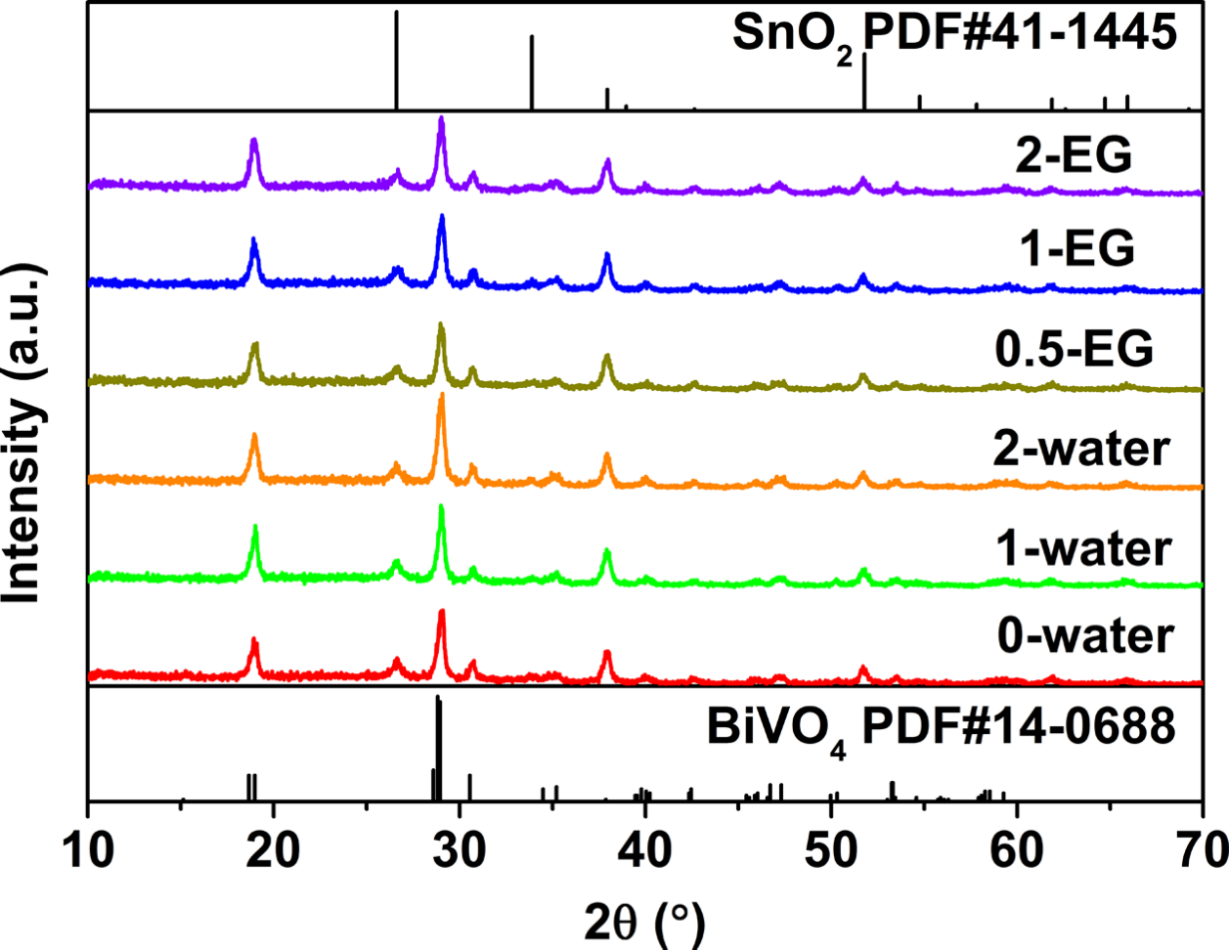
X-ray diffraction patterns of as prepared W-doped BiVO_4_ films.

X-ray diffraction was used to characterize the crystal structure of the obtained films. Figure 2 shows that all peaks agree well with the ones of BiVO_4_ (PDF#14-0688). No peaks belonging to other phases were present except the ones from the fluorine-doped tin oxide (SnO_2_) substrate. This demonstrates BiVO_4_ thin films can be successfully synthesized by the sol–gel method. Elemental analysis was also performed to confirm the composition. To simplify the test, two typical samples, 0-water and 1-EG, were selected for the EDX analysis. The results were shown in Table S1 and Table S2 of Supporting Information File 1. Both samples have almost the same composition, the Bi/V/W/O ratio is 1:0.88:0.03:3.45 for the 0-water sample, and Bi/V/W/O is 1:0.88:0.035:3.4 for the 1-EG sample. The stoichiometric ratio agrees with the one for BiVO_4_, and the dopant concentration is about 3%.

**Figure 3:**
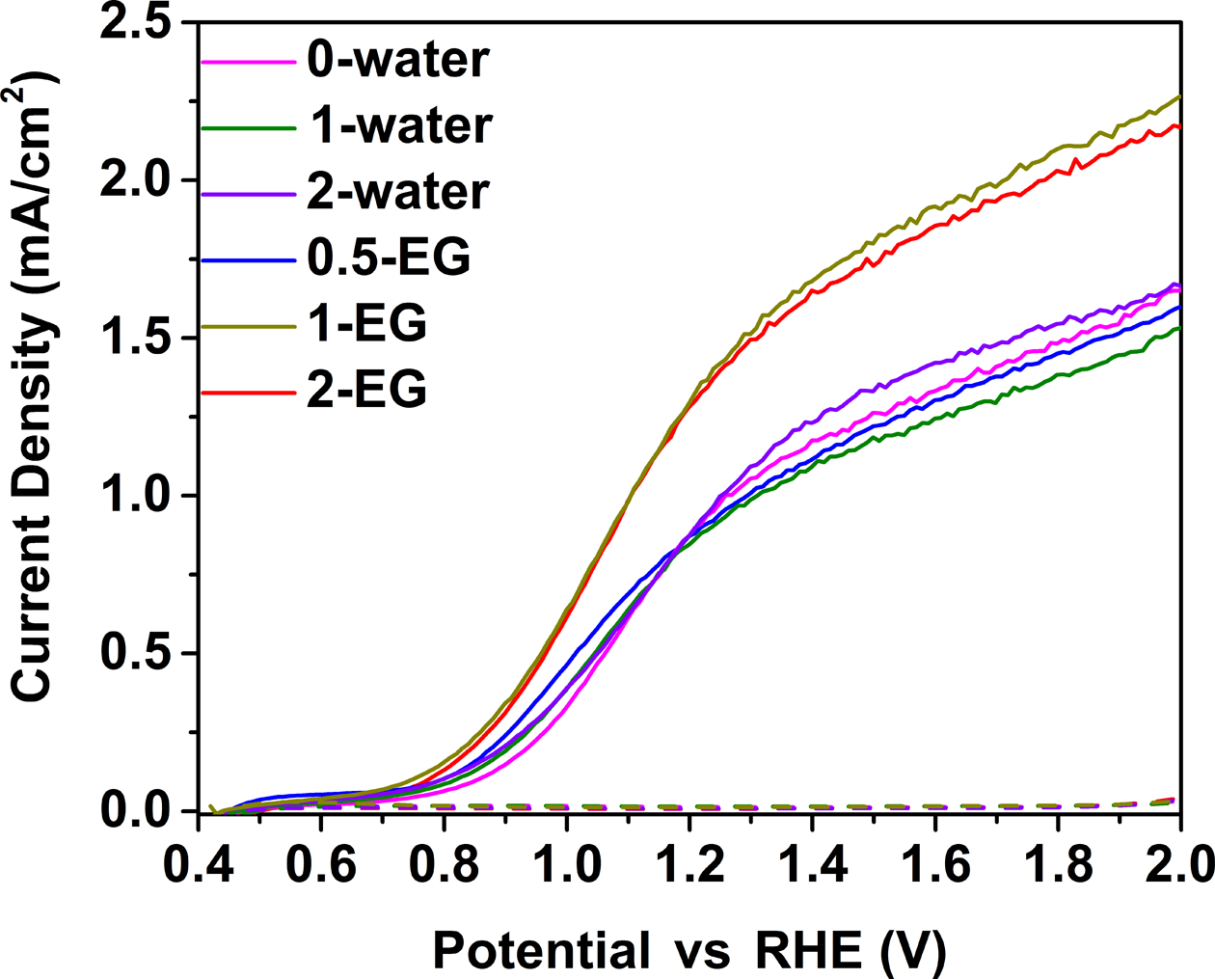
Photocurrents of W-doped BiVO_4_ thin films with different ratios between water and EG.

Figure 3 shows the photocurrents of W-doped BiVO_4_ photoanodes with different morphologies according to Figure 1. The samples prepared using EG precursors have lower photocurrents (around 1 mA/cm^2^ at 1.23 V vs RHE). This is because they mainly display a planar structure (Figure 1a–c), while the samples prepared using water precursor have a nanoporous structure and a better performance (around 1.5 mA/cm^2^ at 1.23 V vs RHE). This corresponds to an increase of ca. 50%. The exception is sample 0.5-EG. As mentioned before, the sample 0.5-EG has poor adhesion and many cracks leading to a poor connection between the sample and the conductive FTO substrate and a worse performance than the other two samples (1-EG, 2-EG).

In the following, we attempt to quantify the contributions from light absorption, charge separation, and charge injection across the electrode/electrolyte interface. These three factors contribute to the water oxidation photocurrent, which is expressed by Equation 1 [21],

[1]
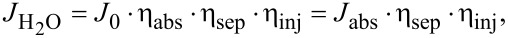


where *J*_H2O_ is the total water splitting photocurrent, *J*_0_ is the theoretical solar photocurrent assuming that all solar energy corresponding to the band edge can be fully converted to fuel energy (7.3 mA/cm^2^ for BiVO_4_), η_abs_ is the light absorption efficiency, η_sep_ is the charge separation efficiency for photo-generated electrons and holes, and η_inj_ is the interfacial charge injection efficiency for water oxidization. *J*_abs_ is the maximum photocurrent of a given photoanode based on its light absorption efficiency.

**Figure 4:**
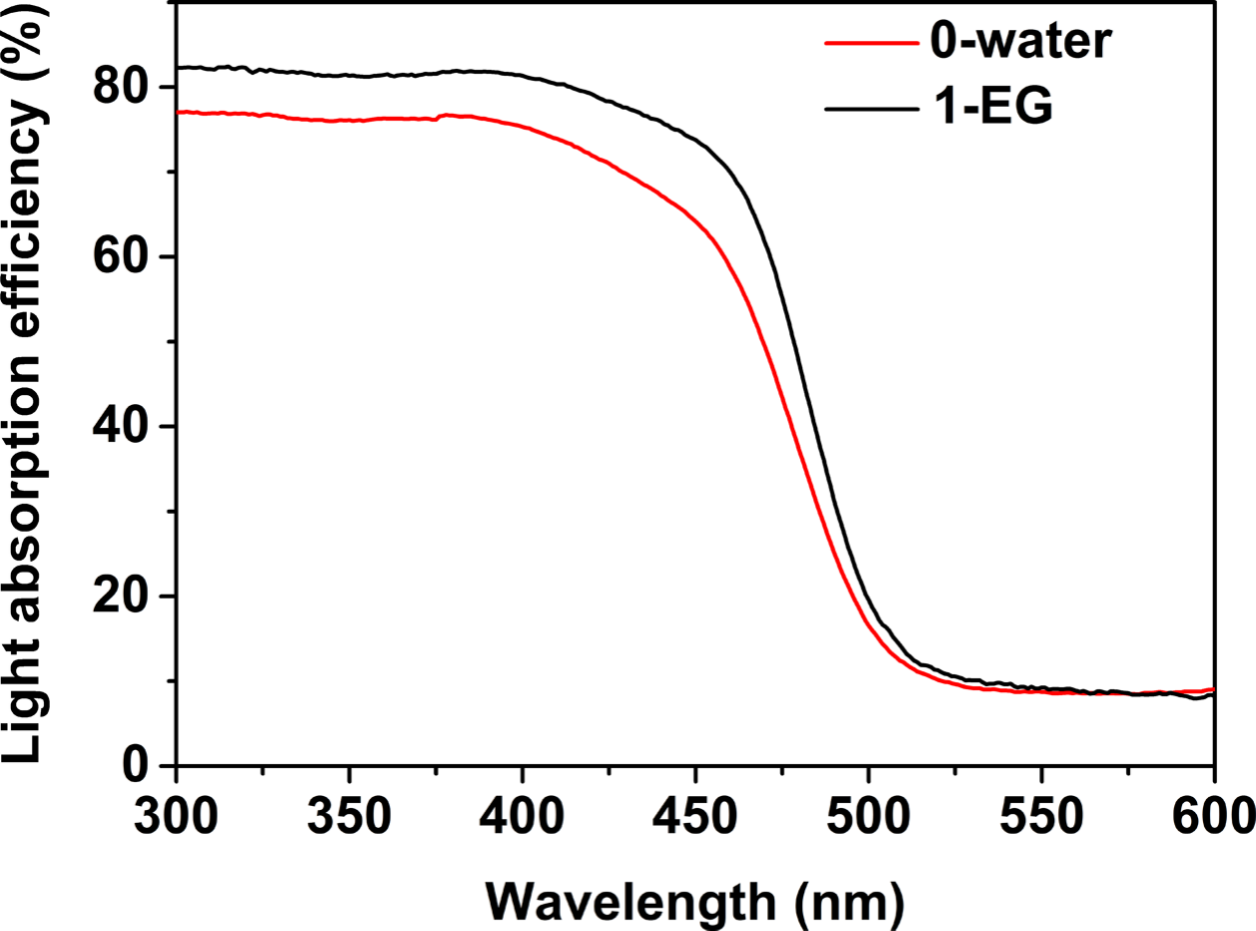
Light absorption efficiency of W-doped BiVO_4_ with planar (0-water) and nanoporous structure (1-EG).

In order to quantify the contributions of the nanostructured W-doped BiVO_4_ in comparison with the planar structure, two representative samples with planar and nanoporous structure, 0-water and 1-EG, were selected. Figure 4 shows the light absorption efficiency of the samples 0-water and 1-EG by measuring the light transmittance and reflectance [12]. The porous structure indeed enhances the light absorption efficiency compared with the planar structure. According to the light absorption efficiencies, *J*_abs_ is calculated to be 3.86 mA/cm^2^ and 4.38 mA/cm^2^ for planar and porous W-doped BiVO_4_, respectively. This means that light absorption enhancement due to the porous structure contributes a 13% increase to *J*_abs_.

The oxidation of water is known to have slow oxidation kinetics. To probe the photoelectrochemical properties, an effective hole scavenger, Na_2_SO_3_, was added to the electrolyte. The interfacial charge injection efficiency can be approximated to be 100% (η_inj_ = 1) due to the fast oxidation kinetics of Na_2_SO_3_ [21]. Under such an approximation, the photocurrent measured with Na_2_SO_3_ electrolyte can be determined by [21]:

[2]
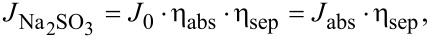


where *J*_Na2SO3_ is the oxidation photocurrent using Na_2_SO_3_. From Equation 1 and Equation 2, we obtain the charge separation efficiency η_sep_ = *J*_Na2SO3_/*J*_abs_ and the charge injection efficiency η_inj_ = *J*_H2O_/*J*_Na2SO3_.

**Figure 5:**
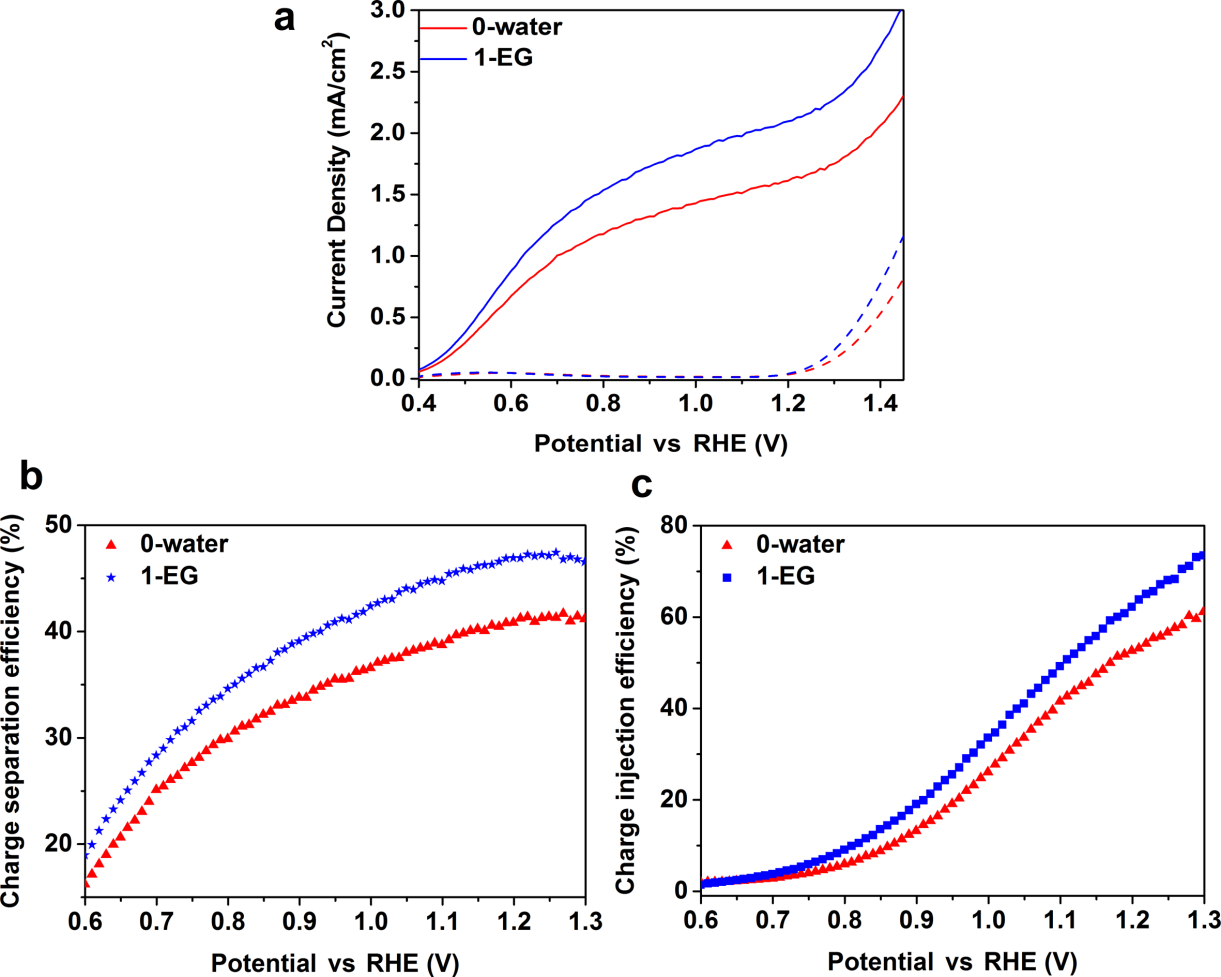
(a) Photocurrents of the samples 0-water and 1-EG measured with hole scavenger Na_2_SO_3_. Dark currents are shown as dashed lines. (b) Charge separation efficiency and (c) charge injection efficiency of the samples 0-water and 1-EG.

Figure 5a shows the oxidation photocurrent density of Na_2_SO_3_ of the sample 1-EG with porous structure at 1.23 V vs RHE (2.06 mA/cm^2^) is 30% higher than that of the planar sample 0-water (1.58 mA/cm^2^). The calculated values of η_sep_ and η_inj_ of the planar and porous W-doped BiVO_4_ are shown in Figure 5b and 5c. η_sep_ at 1.23 V vs RHE of the planar and porous W-doped BiVO_4_ are, respectively, 41% and 47%, representing a 14% increase by the nanostructure formation. η_inj_ at 1.23 V vs RHE of the planar and porous W-doped BiVO_4_ are, respectively, 55% and 66%, corresponding to a 20% increase by the nanostructure formation. It has been reported that porous structures can shorten the hole diffusion distance to the surface and, thus, enhance the charge separation efficiency close to 60% at 1.23 V vs RHE [12]. Our finding is consistent with the report.

**Figure 6:**
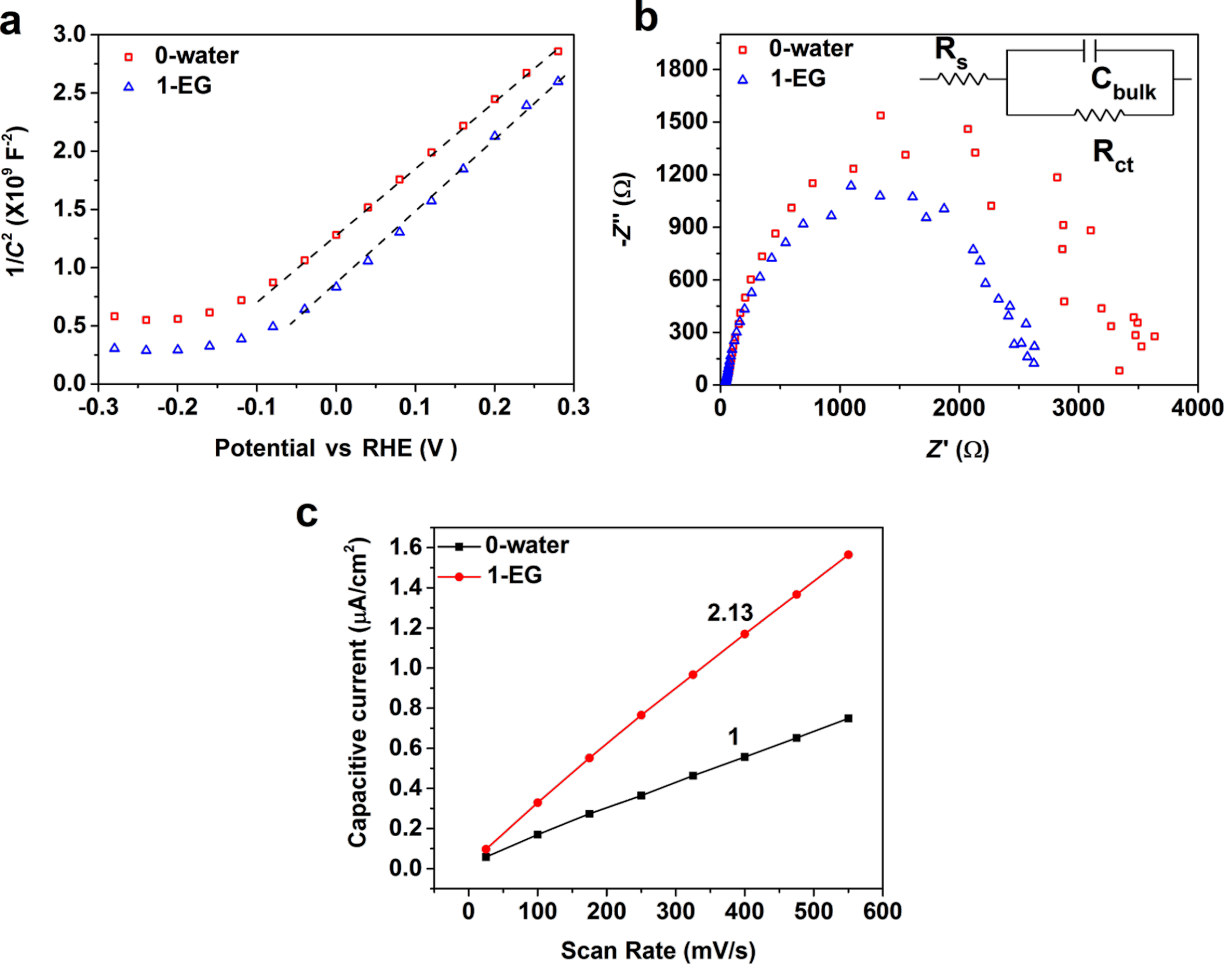
(a) Mott–Schottky plots of W-doped BiVO_4_ with planar (0-water) and nanoporous (1-EG) structure measured at the frequency of 1 kHz in 0.5 M Na_2_SO_4_ aqueous solution. (b) Electrochemical impedance spectra of W-doped BiVO_4_ with planar (0-water) and nanoporous (1-EG) structure at the applied potential of 1.23 V vs RHE under simulated solar illumination in 0.5 M Na_2_SO_4_ electrolyte. Inset is the equivalent circuit employed to fit the experimental EIS data. (c) Relative electrochemical surface area.

Mott–Schottky plots of W-doped BiVO_4_ with planar (0-water) or nanoporous structure (1-EG) were obtained to investigate the carrier density (Figure 6a). The positive Mott–Schottky slopes indicate electrons as the majority carriers. The carrier density can be estimated by Equation 3 [22]:

[3]
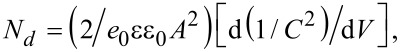


where *e*_0_ is the electron charge (1.60·10^−19^ C), ε is the dielectric constant of BiVO_4_ (68) [23–24], ε_0_ is the electrical permittivity of vacuum (8.85·10^−12^ F·m^−1^), *A* is the electrode area, *N*_d_ is the donor density, *V* is the potential applied at the electrode, and C is the surface capacitance calculated from the electrochemical impedance measured in the dark. The carrier densities of W-doped BiVO_4_ with planar (0-water) and nanoporous (1-EG) structure were found to be 3.7·10^20^ cm^−3^ and 3.3·10^20^ cm^−3^, respectively. This indicates that the structure difference has little effect on the carrier densities, which is understandable since the doping content of W is the same for both samples.

Figure 6b shows the Nyquist plots of W-doped BiVO_4_ with planar (0-water) and nanoporous (1-EG) structure at the applied potential of 1.23 V vs RHE, which provides information on the charge injection process at the interface. Only one semicircle was observed for both samples, and the radius of the semicircle of the planar sample is larger than that of the nanoporous sample. This indicates that the planar sample has a larger surface resistance. According to a previous report, an equivalent circuit is taken to analyze the surface charge injection (inset in Figure 6b) [25]. In the equivalent circuit, *R*_s_ represents the sum of resistance values of the FTO film, the external electrical contacts, and the liquid electrolyte; *R*_ct_ and *C*_bulk_ represent, respectively, the direct charge transfer resistance, and a capacitance at the semiconductor/electrolyte interface. The value of *R*_s_, around 40 Ω, is independent of the film structure. The capacitance of the sample 0-water with planar structure is about 2.27·10^−5^ F, while that of the porous sample 1-EG is about 3.21·10^−5^ F. The difference in the surface capacitance indicates that porous structure provides more surface area, about 2.13-times that of the planar structure (Figure 6c). This is determined based on the capacitive region of the cyclic voltammograms (Figure S3, Supporting Information File 1) according to Jaramillo’s method [26]. The nanoporous structure forms more surface depletion layers, which leads to a higher capacitance. The surface charge transfer resistance of the sample 0-water is about 3783 Ω, while the one of sample 1-EG is smaller at around 2629 Ω. The smaller charge transfer resistance leads to a higher interfacial charge injection efficiency for water oxidation as shown in Figure 5c. It was reported that a porous film with a larger surface area has led to a lower interfacial charge transfer efficiency for water oxidation than its dense counterpart [12]. The current work indicates an opposite trend in that the surface charge injection was improved by the formation of the nanoporous structure. This reason for such an opposite tread remains unknown, and requires further work in the future. We suspect different synthesis methods may have a greater influence on the surface states, which leads to different charge transfer efficiencies.

## Conclusion

Morphologically controlled W-doped BiVO_4_ films with planar and porous structures were prepared by simply tuning the solvent composition during the preparation of the precursor solution. A nearly 50% photocurrent enhancement has been observed due to the formation of the porous structure. The porous structure enhances the light absorption as well as the charge separation due to the short hole diffusion path to the surface. The porous structure also provides more surface reaction sites, estimated to be ca. two times that of the planar film. The surface charge transfer resistance of W-doped BiVO_4_ has been found to decrease for the porous film compared with the planar one, which leads to an enhancement in the interfacial charge injection efficiency. The quantitative analysis shows that three factors contribute to the photoelectrochemical performance enhancement: 13% from enhanced light absorption, 14% from improved charge separation and 20% from increased interfacial charge injection.

## Experimental

**Sample preparation:** Bismuth trioxide, ammonium metavanadate and ammonium tungstate hydrate were dissolved in water or ethylene glycol (EG) with proper amounts of nitric acid to form 0.1 M precursor solutions of Bi, V and W in water or EG. The first three samples were prepared using precursor solutions of Bi, V and W in EG. The solutions were mixed according to the stoichiometric ratio (Bi/V/W = 100:97:3 corresponding to volume a ratio of 500 µL:485 µL:15 µL) for 3% W-doped BiVO_4_. Citric acid (CA) was also added according to a stoichiometric ratio of CA/M = 1.5:1 (M is the total amount of cations). To the mixture different amounts of water or EG were added as follows: to sample 1 0 mL water and 2 mL EG were added (denoted as 0-water); to sample 2 1 mL water and 1 mL EG solution were added (denoted as 1-water); to sample 3 2 mL water and 0 mL EG solution were added (denoted as 2-water).

Samples 4–6 were prepared using precursors solutions of Bi and V in water and of W in EG. The solutions were mixed first, with a volume ratio of Bi/V/W = 500 µL:485 µL:15 µL. Similar to the case above, to the mixture different amounts of water or EG were added: to sample 4 1.5 mL water and 0.5 mL EG were added (denoted as 0.5-EG); to sample 5 1 mL water and 1 mL EG were added (denoted as 1-EG); to sample 6 0 mL water and 2 mL EG were added (denoted as 2-EG). Detailed information is provided in Table 1. After mixing, 60 µL of the precursor solution were dropped on 1 × 1 cm^2^ FTO substrates (1 cm × 2 cm with half of the length covered by thermal tape). The samples were dried at 120 °C for 30 min, and after tearing off the tape the films were subsequently calcined at 500 °C for 2 h in a furnace.

**Characterization:** The morphologies were observed using a field-emission scanning electron microscope (FESEM, JEOL JSM-7600F). Crystallinity was identified by X-ray diffraction (XRD) patterns (Shimadzu 6000 X-ray diffractometer) with Cu Kα radiation (λ = 0.154 nm), using a 2θ scan mode with a fixed incidence angle at 5°. Elemental analysis was conducted by energy-dispersive X-ray spectroscopy (EDX) equipped on a field-emission scanning electron microscopy (FESEM, JEOL JSM-7600F). Light absorption was measured using a UV–visible spectrophotometer by measuring the reflectance and transmittance with an integrating sphere (Lambda 950, Perkin-Elmer). Photoelectrochemical performance was evaluated using a three-electrode configuration (PCI4/300™ potentiostat with PHE200™ software, Gamry Electronic Instruments, Inc.), with the W-doped BiVO_4_ thin film as the working electrode, a Pt foil as a counter electrode, and a Ag/AgCl electrode as a reference electrode. The light source for photoelectrochemical water splitting measurement is a solar simulator (HAL-320, Asahi Spectra Co., Ltd.) with a power intensity of 100 mW·cm^−2^. The photocurrent was measured in a 0.5 M Na_2_SO_4_ aqueous solution with a scan rate of 30 mV·s^−1^. The photocurrents with hole scavenger was measured in a 0.5 M Na_2_SO_4_ aqueous solution with 0.1 M Na_2_SO_3_. Electrochemical impedance spectroscopy (EIS) measurements were carried out under illumination of an AM 1.5G solar simulator in a 0.5 M Na_2_SO_4_ electrolyte at the applied potential of 1.23 V vs RHE using a PCI4/300™ potentiostat (Gamry Electronic Instruments, Inc.). The Mott–Schottky measurements were carried out using an AUTOLAB Potentiostat-Galvanostat (AUTOLAB PGSTAT302 N) at a fixed frequency of 1 kHz in 0.5 M Na_2_SO_4_ aqueous solution.

## Supporting Information

Supporting Information features photocurrent measurements of pristine BiVO4, an SEM image of the sample 0.5-EG, cyclic voltammograms of porous and planar films, and elemental analyses of the samples 0-water and 1-EG by EDX.

File 1 Additional experimental data.
