# Supplementary material for: Enhanced photoelectrochemical water splitting performance using morphology-controlled BiVO4 with W doping
Source: Beilstein J Nanotechnol. 2017 Dec 7;8:2640–7. doi: 10.3762/bjnano.8.264 (PMC5727771; doi:10.3762/bjnano.8.264)
Supplement: File 1 — Additional experimental data. [file Beilstein_J_Nanotechnol-08-2640-s001.pdf]

Supporting Information

for

Enhanced photoelectrochemical water splitting performance using morphology-controlled $BiVO_4$ with W doping

Xin Zhao and Zhong Chen*

Address: School of Materials Science and Engineering, Nanyang Technological University,

50 Nanyang Avenue, 639798, Singapore, Singapore

Email: Zhong Chen - ASZChen@ntu.edu.sg

* Corresponding author

Additional experimental data

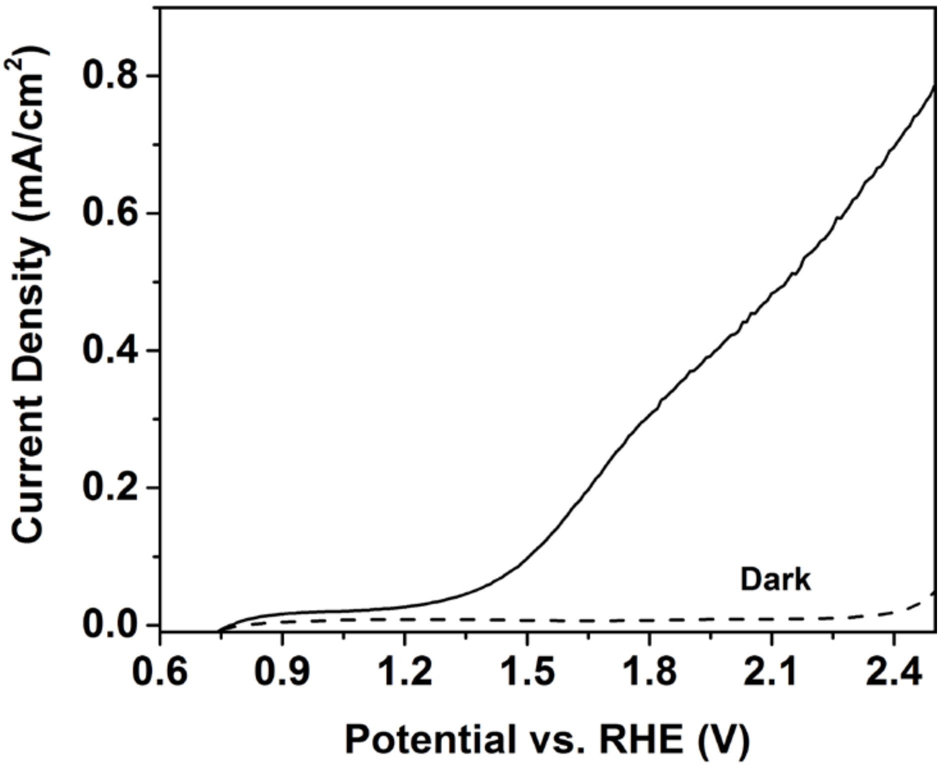

Figure S1: Photocurrent of pristine $BiVO_4$ under AM 1.5G solar light.

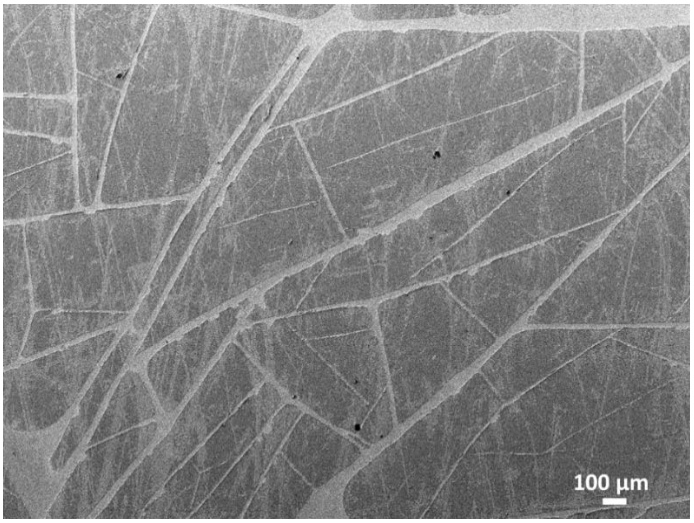

Figure S2: SEM image of 0.5-EG sample at low magnification.

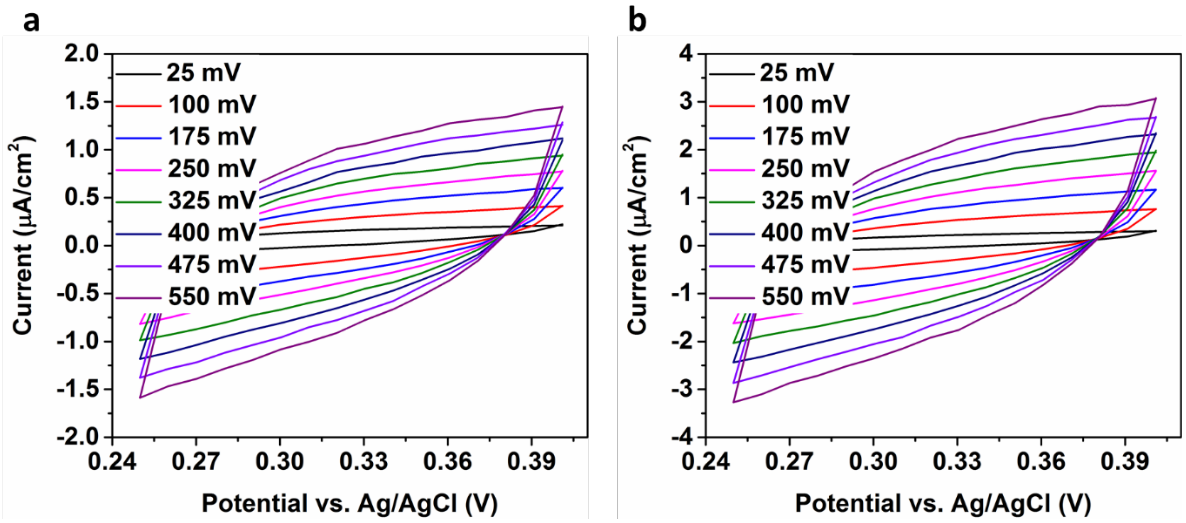

Figure S3: Cyclic voltammograms of (a) W doped $BiVO_4$ with planar (0-water) and (b) nanoporous structure (1-EG) measured at different scan rates from 25 to 550 $mV \cdot s^{-1}$.

Table S1: Elemental analysis of 0-water sample by EDX.

Element	Intensity	Wt %	Atom %
O	0.65	17.42	64.14
V	0.96	14.31	16.55
Bi	0.91	66.46	18.73
W	0.78	1.80	0.58
Total		100	

Table S2: Elemental analysis of 1-EG sample by EDX.

Element	Intensity	Wt %	Atom %
O	0.65	17.28	63.91
V	0.96	14.32	16.64
Bi	0.91	66.35	18.79
W	0.79	2.06	0.66
Total		100	
